# Association Between Waist Circumference and Coronary Artery Disease: Evidence from the NHANES 1999–2023 Cross-Sectional Data and Mendelian Randomization Analysis

**DOI:** 10.5334/gh.1529

**Published:** 2026-02-26

**Authors:** Liheng Chen, Qian Shang, Yu Li

**Affiliations:** 1 Department of Cardiac Surgery, Wuhan Asia Heart Hospital, Wuhan University of Science and Technology, Wuhan, 430022, Hubei, P.R. China

**Keywords:** Waist Circumference, Coronary Artery Disease, Mendelian Randomization, Central Obesity, Propensity Score Matching

## Abstract

**Background:**

Central obesity, as indicated by waist circumference (WC), is a major risk factor for coronary artery disease (CAD). However, the independent causal role of WC in CAD remains underexplored, particularly after adjusting for metabolic comorbidities such as hypertension and diabetes.

**Objectives:**

This study aims to evaluate the causal relationship between WC and CAD using a two-pronged approach: propensity score-matched observational analysis and Mendelian randomization (MR) analysis.

**Methods:**

Data from the National Health and Nutrition Examination Survey (NHANES) 1999–2023 were used for cross-sectional analysis, while genetic instrumental variables associated with WC were sourced from genome-wide association studies (GWAS). We performed inverse variance weighted (IVW) MR analysis and sensitivity tests including MR-Egger and leave-one-out analysis.

**Results:**

Propensity score matching showed that WC was significantly higher in the CAD group compared to controls (*p* < 0.001). MR analysis confirmed a causal relationship between increased WC and CAD risk, with an estimated causal effect size of 0.02884 (95% CI: 0.016, 0.041; *p* = 0.00000883). Sensitivity analyses validated the robustness of these findings.

**Conclusion:**

Our results provide strong genetic and observational evidence linking increased WC with a higher risk of CAD. These findings highlight the need for targeted interventions to reduce central obesity and prevent CAD, especially in populations prone to metabolic disorders.

## Introduction

Obesity and its associated metabolic syndromes are critical public health challenges worldwide, significantly contributing to the burden of chronic diseases (1). Obesity is a well-established risk factor for coronary artery disease (CAD) (2) and is linked to elevated rates of cardiovascular disease (CVD)-related mortality (3). Although the overall CAD mortality rate in the United States has declined significantly over the past 60 years (4), the prevalence of obesity among adults is projected to reach nearly 50% by 2030 (5). This rising trend suggests that, despite improvements in CAD management, the absolute number of CAD cases and related deaths may not decrease accordingly. As obesity becomes increasingly prevalent, it is crucial to identify specific obesity-related factors that contribute to CAD risk to develop more targeted prevention strategies.

Waist circumference (WC), an important marker of central obesity, has been strongly associated with adverse cardiovascular events (6,7,8). Compared to traditional measures such as body mass index (BMI), WC is a better indicator of visceral fat distribution and a more reliable predictor of CVD risk (9,10). Furthermore, central obesity, as measured by WC, has been found to be more directly associated with CAD mortality than general obesity indices like BMI, highlighting its importance in evaluating CAD risk (11). This association persists even after adjusting for other cardiovascular risk factors, making WC a strong independent predictor of CAD (12). However, while observational studies have consistently reported an association between WC and CAD, their inherent limitations, such as residual confounding and reverse causality, hinder the establishment of a clear causal relationship (13).

Several studies, including ‘Individual and Neighborhood Influences on the Relationship Between Waist Circumference and Coronary Heart Disease,’ have attempted to address these limitations by adjusting for comorbid conditions such as hypertension, diabetes, and dyslipidemia rather than excluding these individuals from analysis (14). However, such approaches risk masking the independent impact of central obesity on CAD development, given the complex interplay between these comorbidities and obesity-related metabolic pathways. Similarly, a study applied Mendelian randomization (MR) to establish a causal link between WC and CAD, but the findings the obviously influence of prevalent chronic conditions like hypertension and diabetes on the WC–CAD relationship (15). The other study just showed the sex-specific Mendelian randomization study, cannot stand for whole relationship between the WC and CAD (16). Therefore, the need remains for a study that can eliminate these confounding factors to better understand the direct impact of WC on CAD risk.

Our study aims to address these limitations by combining the strengths of both observational and genetic approaches to provide more robust evidence for the causal role of WC in CAD development. First, we leverage the National Health and Nutrition Examination Survey (NHANES), a nationally representative dataset with diverse racial and ethnic groups, to conduct a comprehensive observational analysis. By excluding individuals with pre-existing hypertension, diabetes, and dyslipidemia, we minimize the influence of these comorbidities, thereby offering a clearer view of the independent relationship between WC and CAD. Furthermore, we employ propensity score matching (PSM) to balance key baseline characteristics (e.g., age, sex, and race) between CAD and non-CAD groups, simulating a randomized controlled trial (RCT)-like setting within an observational framework. This methodological improvement over previous studies, which primarily relied on regression adjustment, enhances the internal validity of our findings by reducing bias from measured confounders.

In addition, we strengthen our findings through Mendelian randomization (MR), a powerful genetic tool that uses genetic variants as instrumental variables to infer causal relationships. Unlike traditional observational studies, MR is less susceptible to confounding and reverse causality, making it ideal for establishing the causal effect of WC on CAD. Although previous MR studies have explored this relationship, they have been limited by their focus on European populations and a lack of consideration for comorbid conditions. Our study uniquely integrates MR and PSM, thereby addressing both genetic and observational limitations. By utilizing a multi-ethnic population from NHANES, we can extend the generalizability of our findings and provide novel insights into how the genetic determinants of WC influence CAD risk across diverse racial and ethnic backgrounds.

This dual-method approach—combining PSM-based observational analysis and MR-based genetic analysis—provides robust evidence that goes beyond the scope of previous studies. By exploring potential genetic-phenotypic interactions in a diverse population, we aim to fill critical gaps in the literature and offer a comprehensive understanding of the independent impact of WC on CAD. Our findings will not only elucidate the causal role of WC in CAD development but also inform targeted intervention strategies for CAD prevention in high-risk populations, making a valuable contribution to the field of cardiovascular research.

## Materials and Methods

### Study design and data source

**Figure 1:**
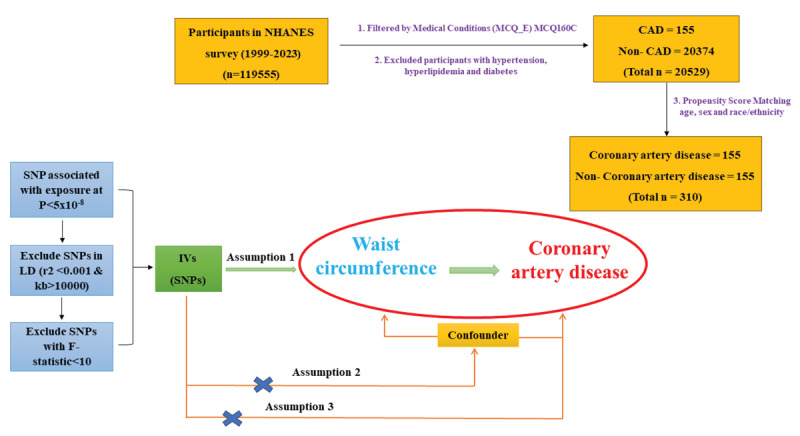
Flowchart of the study process to evaluate the association between waist circumference (WC) and coronary artery disease (CAD) using observational and Mendelian randomization (MR) analyses.

This study used a two-part approach, integrating cross-sectional data analysis and Mendelian randomization (MR) to assess the association between WC and CAD risk (Figure 1).

### Cross-sectional analysis

This study utilized data from the National Health and Nutrition Examination Survey (NHANES, https://www.cdc.gov/nchs/nhanes/index.htm) from 1999 to 2023, which is representative of the non-institutionalized US population, including data from the most recently available cycle (August 2021–August 2023; first release: September 20, 2024). Detailed information on the NHANES recruitment procedures, population characteristics, and study design is publicly available on the Centers for Disease Control and Prevention (CDC) website. For this study, we included only participants who completed a comprehensive physical examination (Body Measures) and a self-reported coronary heart disease questionnaire as part of the NHANES assessments. To minimize confounding effects, individuals with a history of diabetes, hypertension, or hyperlipidemia were excluded from the initial analysis.

To further improve baseline comparability between the CAD and control groups, we performed propensity score matching (PSM) using 1:1 nearest-neighbor matching without replacement and applied a caliper width of 0.2 standard deviations of the logit of the propensity score. This approach was selected to optimize covariate balance—specifically age, sex, and race/ethnicity—while avoiding overfitting and maintaining interpretability. Height, weight, and BMI were excluded from the matching process due to their strong collinearity with waist circumference, which could bias the analysis. Similarly, other variables such as smoking status, liver function, and kidney function were not included in the matching in order to preserve an adequate sample size and maintain statistical power.

Subsequent statistical analyses were conducted using R version 4.4.1. Categorical variables were compared using the χ^2^ test, while continuous variables were assessed using analysis of variance (ANOVA) or the Kruskal–Wallis rank-sum test as appropriate. A two-tailed *p*-value of < 0.05 was considered statistically significant. This study adhered to the Strengthening the Reporting of Observational Studies in Epidemiology (STROBE) guidelines for observational research. Ethical approval was not required for secondary analysis of publicly available, de-identified NHANES data.

### Mendelian randomization analysis

Our manuscript adheres to the STROBE-MR guidelines (https://www.strobe-mr.org/) for the reporting of Mendelian Randomizations. To minimize the potential influence of pleiotropy, we performed additional sensitivity analyses using the MR-PRESSO (Mendelian Randomization Pleiotropy RESidual Sum and Outlier) method, which detects horizontal pleiotropy and corrects for outlier SNPs. Furthermore, each selected SNP was assessed using the PhenoScanner database to ensure that none were associated with known confounders, including BMI, diabetes, hypertension, hyperlipidemia, and other cardiovascular traits. First, the genetic variants selected as IVs for waist circumference was chosen at a genome-wide significance level, ensuring a direct impact on these traits. Second, these genetic variants were confirmed to have no associations with potential confounders. Third, the genetic variants were assumed to influence CAD only through their effects on waist circumference. To identify IVs associated with waist circumference, we conducted a search within the genome-wide association study (GWAS) dataset for CAD. We identified the corresponding single nucleotide polymorphisms (SNPs) linked to waist circumference. SNPs do not present in the outcome GWAS dataset were excluded from further analysis.

We accessed the MR Base database (http://www.mrbase.org/), which contains an extensive collection of summary statistics from numerous genome-wide association studies (GWAS). Specifically, we utilized publicly available summary statistics the UK Biobank GWAS datasets conducted on individuals of European descent total *n* = 462,166 (GWAS ID: ukb-b-9405) as the exposure variables and total *n* = 42,096 (GWAS ID: ebi-a-GCST003116) as the outcome group (available at https://www.ebi.ac.uk/gwas/) (17). The data used in this study were obtained with informed consent from the participants.

To ensure robust inference and strengthen the validity of our findings, we employed a two-sample MR study design, using genetic variants associated with WC as instrumental variables (IVs). These IVs were selected based on their genome-wide significance level, applying a *P*-value threshold of 5 × 10^–8^, and the number of available single nucleotide polymorphisms (SNPs). Additionally, we excluded SNPs in linkage disequilibrium, using a criterion of an *r*^2^ value < 0.001 and a genetic distance of 10,000 KB. The selected IVs were assessed for weak instrument bias by calculating the *F* statistic, which confirmed the strength of the instruments (*F* > 10), indicating no evidence of weak instrument bias.

As a critical step to fulfill the second MR assumption, akin to adjusting for confounders in multivariate analysis, we queried the Phenoscanner database (http://www.phenoscanner.medschl.cam.ac.uk/) to ensure that the included SNPs were not associated with known confounders, such as body mass index, hypertension, hyperlipidemia and diabetes.

The causal relationship between WC and CAD was assessed using several Mendelian randomization (MR) methods, including inverse variance weighted (IVW), weighted median, MR-Egger, and weighted mode. Sensitivity analysis was conducted with the leave-one-out method. The results were visually presented through scatter plots, funnel plots, and forest plots. All analyses were performed on the MR Base online platform (http://app.mrbase.org/) using the ‘TwoSampleMR’ R package (18). A *p*-value of < 0.05 was considered statistically significant, indicating a potential causal effect.

## Results

### Baseline characteristics after propensity score matching

After performing propensity score matching (PSM), a total of 310 participants were included in the analysis, with 155 individuals in the CAD group and 155 in the non-CAD group (selected from 20,374 non-CAD participants) (Table 1). As expected from the matching procedure, the demographic variables of age, sex, and race/ethnicity were well balanced between the two groups. The mean age was nearly identical between the CAD group (68.08 ± 14.59 years) and the non-CAD group (68.12 ± 14.63 years; *p* = 0.978). The gender distribution was also perfectly matched, with 76.1% males and 23.9% females in both groups (*p* = 1.0).

**Table 1:** Baseline characteristics and results after propensity score matching.


	LEVEL	CORONARY ARTERY DISEASE	NON-CORONARY ARTERY DISEASE	*P*

*N*		155	155	

BMXWAIST (mean (SD))		124.50 (14.52)	99.36 (12.65)	<0.001

RIDAGEYR (mean (SD))		68.08 (14.59)	68.12 (14.63)	0.978

RIAGENDR (%)	Male	118 (76.1)	118 (76.1)	1

	Female	37 (23.9)	37 (23.9)	

RIDRETH1 (%)	Mexican American	18 (11.6)	19 (12.3)	0.999

	Other Hispanic	7 (4.5)	8 (5.2)	

	Non-Hispanic Black	10 (6.5)	10 (6.5)	

	Non-Hispanic White	114 (73.5)	112 (72.3)	

	Other Race	6 (3.9)	6 (3.9)	


Similarly, there were no significant differences in the ethnic distribution between the CAD and non-CAD groups (*p* = 0.999). In the CAD group, 11.6% were Mexican American, 4.5% were Other Hispanic, 6.5% were Non-Hispanic Black, 73.5% were Non-Hispanic White, and 3.9% were of Other Race. The non-CAD group showed a comparable distribution: 12.3% Mexican American, 5.2% Other Hispanic, 6.5% Non-Hispanic Black, 72.3% Non-Hispanic White, and 3.9% Other Race.

In contrast, the mean waist circumference (WC) was significantly higher in the CAD group compared to the non-CAD group (124.50 ± 14.52 cm vs. 99.36 ± 12.65 cm, respectively; *p* < 0.001). This suggests that central obesity, as indicated by waist circumference, may be independently associated with CAD risk after controlling for demographic factors. The successful balance of age, sex, and race/ethnicity in both groups confirms that the PSM method effectively controlled for these confounders, allowing a clearer assessment of the independent impact of waist circumference on CAD risk.

### Mendelian randomization analysis results

Mendelian randomization (MR) analysis was conducted to evaluate the causal relationship between waist circumference (WC) and the risk of CAD using genetic instruments (Table 2). A total of 340 independent single nucleotide polymorphisms (SNPs) associated with WC were selected as instrumental variables (IVs) after applying genome-wide significance thresholds (*p* < 5 × 10^–8^) and linkage disequilibrium filtering (*r*^2^ < 0.001, 10,000 kb distance). The primary MR analysis utilized these SNPs from the ukb-b-9405 dataset, while the outcome was derived from the ebi-a-GCST003116 dataset. The MR-PRESSO analysis did not detect any outlier SNPs, indicating that the observed association was unlikely to be influenced by pleiotropy. Additionally, Cochran’s Q test was used to evaluate heterogeneity among SNPs, with a non-significant Q value suggesting homogeneity across genetic instruments.

**Table 2:** Data description of waist circumference and coronary artery disease.


	PHENOTYPE	POPULATION	SAMPLE SIZE	DATE	SNPS	ACCESS ADDRESS

Exposure	Waist circumference	European	462,166	2018	9,851,867	https://gwas.mrcieu.ac.uk/datasets/ukb-b-9405/
	
Outcome	Coronary artery disease		42,096	2015	8,597,751	https://gwas.mrcieu.ac.uk/datasets/ebi-a-GCST003116/


#### Primary MR analysis

The primary Mendelian randomization (MR) analysis using the inverse variance weighted (IVW) method revealed a significant positive association between genetically predicted waist circumference (WC) and coronary artery disease (CAD) risk. The estimated causal effect size was 0.02884 (95% CI: 0.016, 0.041; *p* = 0.00000883), suggesting that increased WC is associated with a higher risk of CAD. The weighted median method provided similar estimates (effect size: 0.03205, 95% CI: 0.012, 0.052; *p* = 0.00127), further reinforcing the robustness of the IVW findings. MR-Egger regression, which accounts for potential horizontal pleiotropy, showed a slightly larger effect size (0.037, 95% CI: 0.005, 0.069) with a larger standard error, indicating the possible presence of pleiotropy. However, the MR-Egger intercept was non-significant (intercept = 0.001, *p* = 0.712), suggesting pleiotropy is unlikely to have substantially biased the results (Table 3).

**Table 3:** The results about MR.


EXPOSURE	OUTCOME	METHOD	nSNP	BETA	SE	*p*-VALUE

Waist circumference	Coronary Artery disease	MR Egger	340	0.6342	0.1354	4.064e–06

		Weighted median	340	0.5183	0.07244	8.345e–13

		Inverse variance weighted	340	0.497	0.04743	1.084e–25

		Weighted mode	340	0.572	0.1467	0.000116


#### Heterogeneity analysis

Cochran’s *Q* test revealed significant heterogeneity in both the MR-Egger (*Q* = 534.6, *p* = 4.69e–11) and IVW methods (*Q* = 536.4, *p* = 4.184e–11), indicating substantial variability across the SNPs used as instrumental variables. This high level of heterogeneity suggests that the genetic instruments may not be entirely consistent in their effects on WC and CAD risk. Potential explanations for this heterogeneity include the presence of pleiotropic effects or variability in the direction of SNP effects. Despite this, the MR-Egger intercept was not statistically significant, which reduces concerns about horizontal pleiotropy as a major source of bias.

Although significant heterogeneity was observed, largely due to the diversity of SNP effects, sensitivity analyses and leave-one-out tests demonstrated that this heterogeneity had limited influence on the overall results. While heterogeneity introduces some uncertainty into the model, the findings consistently indicate a significant causal relationship between WC and CAD. Therefore, it can be reasonably inferred that WC is an independent risk factor for CAD, though care should be taken when interpreting the results, considering the potential influence of pleiotropy and heterogeneity.

#### Sensitivity analyses

**Figure 2:**
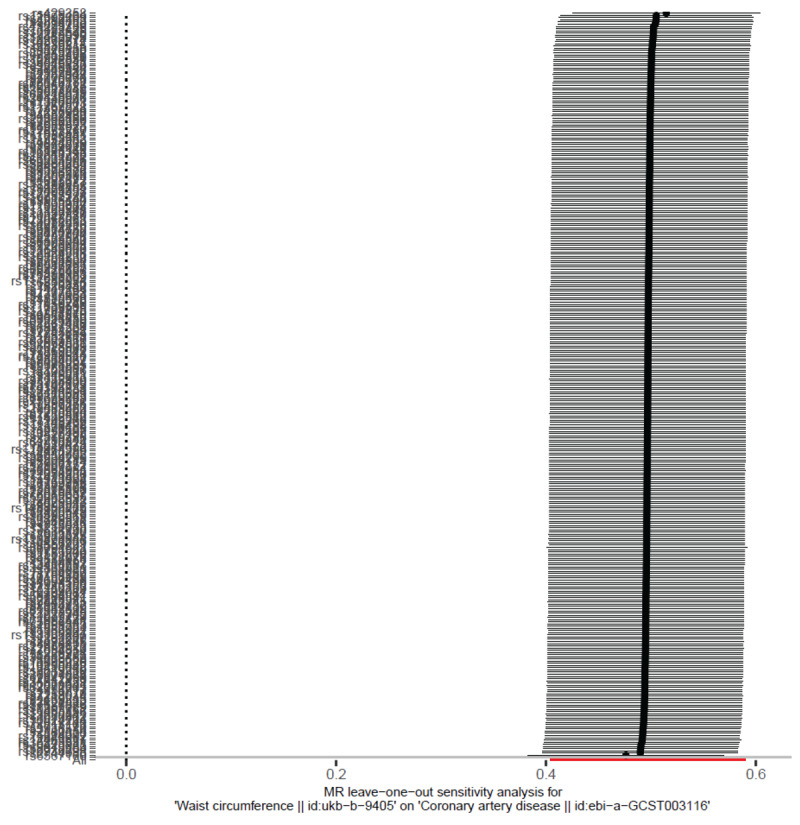
This figure presents the results of the leave-one-out sensitivity analysis, where each SNP is sequentially removed from the analysis to assess its individual influence on the overall causal estimate. The *x*-axis represents the causal effect estimates of waist circumference (WC) on coronary artery disease (CAD) after removing each SNP, while the *y*-axis represents the SNPs. The consistency of the causal effect across different iterations of SNP exclusion indicates that no single SNP disproportionately influences the overall results. This supports the robustness of the causal estimate and minimizes concerns about outlier SNPs driving the observed association.

**Figure 3:**
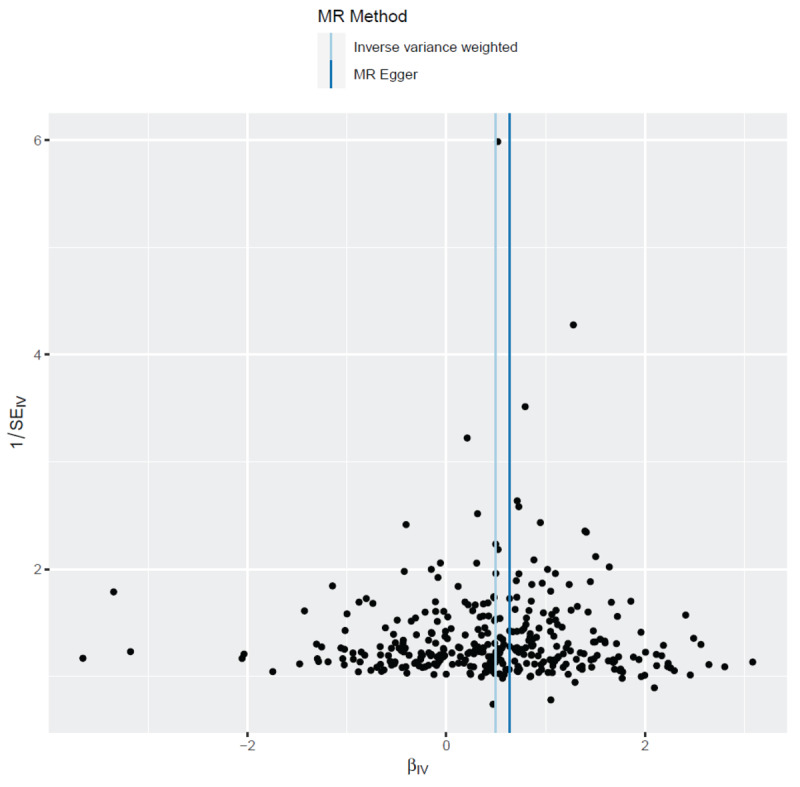
The funnel plot visualizes the distribution of individual SNP effects on the association between WC and CAD. The *x*-axis represents the effect size (log odds ratio) of each SNP, and the *y*-axis shows the standard error of these estimates. The symmetry of the plot suggests that directional pleiotropy is unlikely to be a significant concern in this analysis. A symmetric distribution indicates that the observed effects are not biased by pleiotropic SNPs, supporting the validity of the causal inference.

To further evaluate the robustness of our findings, leave-one-out sensitivity analysis was conducted. This analysis showed that the removal of any single SNP did not materially alter the overall causal estimates, indicating that no single SNP disproportionately influenced the overall results (Figure 2). The funnel plot of SNP effects was symmetric, providing further evidence that directional pleiotropy is unlikely to have affected the findings (Figure 3).

#### Visualization of MR results

**Figure 4:**
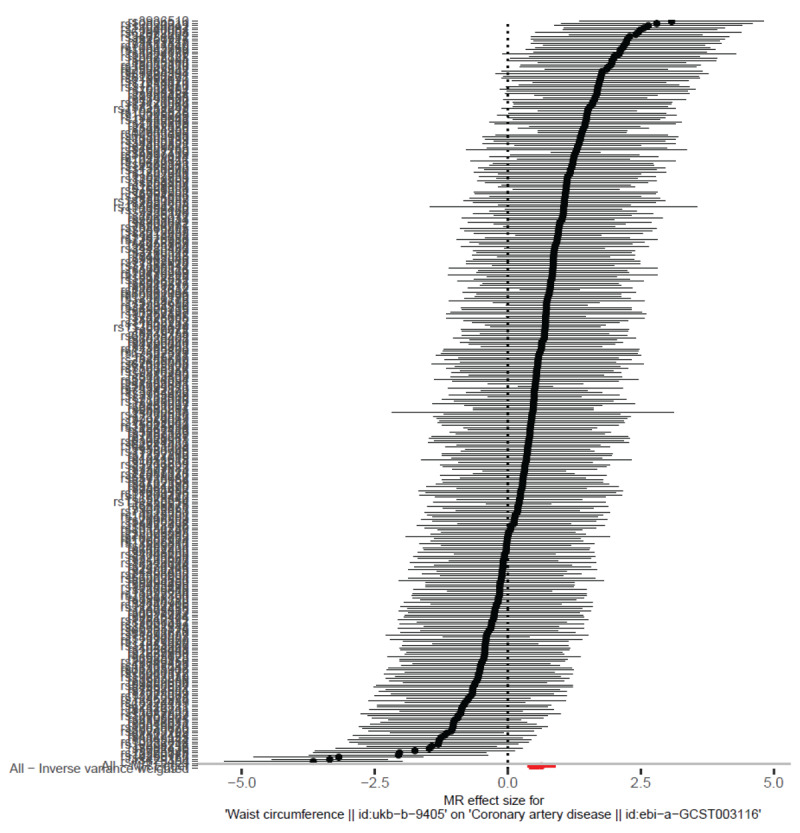
This forest plot displays the individual causal effects of each SNP on CAD risk, with the *x*-axis representing the estimated effect sizes and confidence intervals for each SNP. Most SNPs show a positive association between WC and CAD, with confidence intervals overlapping, supporting a consistent direction of effect. This consistency across SNPs reinforces the overall finding that increased WC is causally associated with a higher risk of CAD.

**Figure 5:**
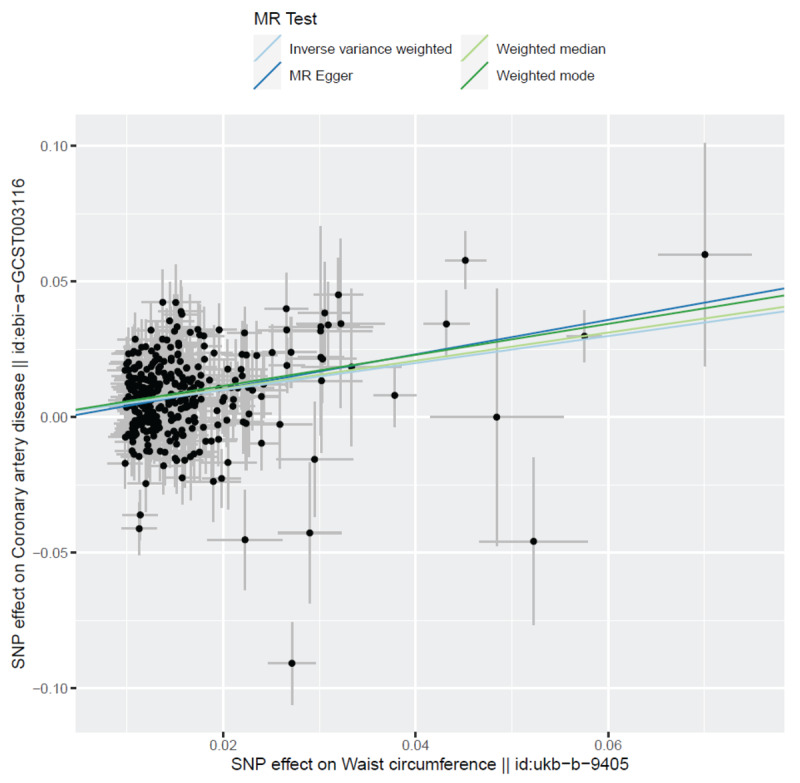
The scatter plot compares the SNP-specific causal estimates across different MR methods, including inverse variance weighted (IVW), MR-Egger, and weighted median. The *x*-axis represents the WC effect size, while the *y*-axis shows the CAD effect size. The alignment of the SNP-specific estimates across different MR methods suggests consistency in the direction and magnitude of the association between WC and CAD. The close overlap of the IVW and weighted median lines supports the robustness of the results, indicating that the causal relationship remains consistent regardless of the MR method used.

The forest plot (Figure 4) illustrates the causal effect of each SNP on CAD risk, with the majority showing a positive association between WC and CAD. This supports the consistency of the direction of effect for most SNPs. The scatter plot (Figure 5) further highlights the alignment of SNP-specific causal estimates across different MR methods, with a strong overlap between the IVW and weighted median models, confirming the robustness of the association.

Despite the presence of significant heterogeneity, the overall findings from multiple MR methods consistently support a causal relationship between increased WC and elevated CAD risk. The robustness of the results, even after accounting for potential pleiotropy and sensitivity analyses, strengthens the evidence for WC as an independent risk factor for CAD. These findings underscore the importance of targeting central obesity in the prevention and management of CAD.

## Discussion

In this Mendelian Randomization (MR) study, we established a robust causal relationship between waist circumference (WC), a primary marker of central obesity, and coronary artery disease (CAD) risk. Central obesity has long been linked to adverse metabolic outcomes, and this study provides strong genetic evidence supporting its independent role in CAD development (19,20,21,22). These findings confirm and strengthen the growing body of literature suggesting that visceral fat accumulation, closely associated with increased WC, plays a critical role in the development of cardiovascular diseases.

### Metabolic dysfunction and inflammation

A key mechanism explaining the WC–CAD association is the contribution of visceral adipose tissue to systemic inflammation. Visceral fat actively secretes pro-inflammatory cytokines, including interleukin-6 (IL-6) and tumor necrosis factor-α (TNF-α), both implicated in the development of atherosclerosis and endothelial dysfunction (23,24,25). Elevated levels of these inflammatory markers in individuals with central obesity directly link inflammation to CAD progression.

### Insulin resistance

Another well-established mechanism is the relationship between central obesity and insulin resistance. Visceral fat accumulation is closely tied to insulin resistance, a condition that exacerbates metabolic syndrome and increases CAD risk (26,27,28). Insulin resistance contributes to endothelial dysfunction, heightens vascular inflammation, and promotes atherogenic lipid profiles, such as elevated triglycerides and reduced high-density lipoprotein (HDL) cholesterol-key factors in CAD pathogenesis (29,30,31).

### Atherogenic dyslipidemia

Atherogenic dyslipidemia is another metabolic abnormality common in individuals with central obesity. This lipid profile, characterized by elevated triglycerides, low HDL cholesterol, and small dense low-density lipoprotein (LDL) particles, accelerates the formation of atherosclerotic plaques (1,32,33,34). The relationship between WC and atherogenic dyslipidemia, consistently observed in observational studies and genetic analyses, further supports the causal link to CAD risk.

### Hormonal dysregulation

Although the role of hormones such as leptin and adiponectin in cardiovascular disease is still being investigated, evidence suggests that these hormones, dysregulated in central obesity, may influence CAD risk (35,36,37). Elevated leptin levels have been associated with increased sympathetic activity and arterial stiffness, both of which contribute to CAD (38). Conversely, reduced adiponectin levels, which possess anti-inflammatory properties, have been linked to endothelial dysfunction and heightened atherosclerosis risk (39,40). However, further research is necessary to fully understand the hormonal pathways involved.

In summary, the causal relationship between WC and CAD is likely mediated by multiple mechanisms, including systemic inflammation, insulin resistance, and atherogenic dyslipidemia. These findings align with current understanding of the role of central obesity in metabolic and cardiovascular diseases. Thus, our study carries significant clinical implications, especially regarding public health strategies aimed at reducing central obesity to mitigate the burden of cardiovascular diseases.

### Limitations and future directions

Despite the robust nature of our findings, there are several limitations that should be acknowledged. First, the study sample was limited to individuals of European descent, which restricts the generalizability of the results to other ethnic groups. Future research should aim to replicate these findings in more ethnically diverse populations, as the effects of central obesity and its metabolic consequences may vary across different genetic backgrounds. Second, although physical activity is a well-recognized factor influencing both waist circumference and cardiovascular risk, we were unable to include it in our analysis due to inconsistent or missing data across NHANES cycles. This may have introduced residual confounding in the observational analysis. We now explicitly acknowledge this limitation and recommend that future research incorporate objectively measured physical activity data to improve causal inference. Additionally, although our MR analysis accounted for pleiotropy using multiple sensitivity analyses including MR-Egger and MR-PRESSO, the possibility of residual pleiotropy influencing the results remains. Further research should focus on elucidating the molecular mechanisms linking WC to CAD and validating these findings in longitudinal cohort studies. This would not only strengthen the causal evidence but also provide insights into the temporal relationships between central obesity and CAD risk.

### Mechanistic relevance of genetic instruments

Importantly, many of the SNPs used in our MR analysis were located near biologically relevant loci such as FTO (41,42) and MC4R (43,44), which regulate appetite and energy homeostasis, and TMEM18 (45), which influences fat distribution. These genes are well established in the literature as contributors to central adiposity and metabolic dysfunction, supporting the mechanistic plausibility of our genetic findings and reinforcing the causal role of WC in CAD development.

## Conclusion

This study provides strong genetic evidence for a causal relationship between increased WC and CAD risk, likely mediated through systemic inflammation, insulin resistance, and atherogenic dyslipidemia. Despite heterogeneity in the analysis, sensitivity tests confirmed the robustness of the findings. These results underscore the importance of targeting central obesity in reducing the global burden of CAD. Future research should validate these findings in more diverse populations and further explore the biological mechanisms underlying the WC–CAD relationship.
